# On the brink between extinction and persistence

**DOI:** 10.1186/1745-6150-3-47

**Published:** 2008-11-19

**Authors:** Cino Pertoldi, Lars A Bach, Volker Loeschcke

**Affiliations:** 1 Department of Biology, Ecology and Genetics, University of Aarhus, Ny Munkegade, Bldg. 1540, DK-8000, Aarhus C, Denmark; 2 Mammal Research Institute, Polish Academy of Sciences, Waszkiewicza 1c, 17-230 Białowieża, Poland; 3 Department of Theoretical Ecology, Ecology Building, Lund University, SE-22362 Lund, Sweden

## Abstract

The nature of size fluctuations is crucial in forecasting future population persistence, independently of whether the variability stems from external forces or from the dynamics of the population renewal process. The risk of intercepting zero is highly dependent on the way the variance of the population size relates to its mean. The minimum population size required for a population not to go extinct can be determined by a scaling equation relating the variance to the arithmetic mean. By the use of a derived expression for the harmonic mean defined by the parameters of the scaling equation we show how it is possible to separate the domains of persistence from those of extinction and to facilitate the identification of populations on the brink of extinction.

This article was reviewed by Mark W. Schwartz (nominated by Peter Olofsson), Josef Bryja (nominated by Aniko Szabo) and Wai-YuanTan. For the full reviews, please go to the Reviewers' Comments section.

## Background

Natural populations are affected by σ^2^_e _and σ^2^_d _which in turn affect the expected time to extinction [1]. Although the time to extinction is expected to increase with population size [2], other factors influence the dynamics of populations as e.g. density dependent mechanisms and population growth rate [3,4]. The specific population growth rate is affecting the long-term persistence of populations, whereas the most immediate effects on the risk of extinction are mainly due to σ^2^_e_. Especially in small populations, population persistence is affected by σ^2^_d _[5]. Reproduction and survival of offspring can be dependent or independent of population size and in combination cause populations to fluctuate. Moreover, recent work points to the importance of altered σ^2^_e _on the variation of vital rates, which obviously feed-back on the demographic dynamics [6]. Studies developing methods for estimating population viability are growing steadily [7,8].

## Presentation of hypothesis

Here we propose a simple model to estimate the risk of extinction and population persistence based upon a two-parameter description of the harmonic mean (HM), defined by the parameters of the scaling equation [9]. This method allows a separation of the domains of population persistence *versus *those of extinction and enables the identification of populations on the brink of extinction as it allows the estimation of the minimum population size required for population persistence. The HM has the special property that a single occurrence/generation of zero suffices to cause ultimate population extinction – much like the behaviour of real populations in the absence of migration.

## Testing the hypothesis

Population fluctuations are influencing the renewal process in a way that simultaneously affect the $\bar{\mu }$ and *σ*^2 ^of N. Taylor's power law gives an expression of how *σ*^2 ^relates to size of $\bar{\mu }$[9]

(1) $$\sigma^{2}=K{\bar{\mu }}^{\beta },$$

where *K *is a measure of individual level variability, and *β *is the scaling exponent. Taylor's power law is well documented for animal populations and suggests that increasing σ^2^_d _increases individual reproductive variance while increasing σ^2^_e _increases reproductive covariance. For populations experiencing constant *per capita *σ^2^_e_, the regression of log σ^2 ^*versus *log $\bar{\mu }$ gives a line with a slope of 2 for trivial mathematical reasons. Data from time series of natural populations suggest that *β *may lie anywhere in the range of 0.6 to 2.8 [10,11]. The range of *K *has been estimated empirically in many populations to lie in the range 0.10 <*K *< 8.32 [10,11].

The degree of reproductive covariance among individuals affects the scaling exponent *β*. Completely correlated reproduction results in *β *= 2, while independent reproduction results in *β *= 1 [12]. Organisms that have highly correlated responses to environmental fluctuations will exhibit less variable reproductive patterns than organisms that experience a high degree of σ^2^_e _on the individual scale.

Population size is expected to follow a log-normal distribution (which becomes normally distributed if log-transformed), given that it is the product of temporally multiplicative renewal processes [13-15]. The assumption of the model (a normal or log-normal distribution) is valid both for populations under exponential growth or decline and for populations at equilibrium [13]. It has been demonstrated in a survey of 544 long-term time series of terrestrial and aquatic organisms that about one-half of them are log-normally distributed, which implicates that our model can be applied also to long-term time series [15]. Under the assumption of normal or log-normally distributed population fluctuations, it can be shown that HM of the population size series relates to the size of $\bar{\mu }$ through:

(2) $$HM=\bar{\mu }-K{\bar{\mu }}^{\left(\beta -1\right)}$$

HM can be considered a proxy for N_E_, especially for populations with discrete generations, whereas when there are overlapping generations, the time scale becomes crucial and must be defined such that it relates to the discrete models. Applying the power law to HM allows us to study the separation of domains of attraction to zero from domains of non-zero HM. Subsequently, these domains can be interpreted in terms of persistence and extinction of populations and hence help identifying populations destined for extinction. To explore the domains we investigated various combinations of the parameters *β*, *K *and $\bar{\mu }$. The consequences of *β *> 2 have been discussed earlier [9], and it has been found that HM, for certain values of *β *and K, first increases with increasing $\bar{\mu }$ but then starts to decrease for higher $\bar{\mu }$.

From equation (2) it follows that:

(3) $$0<\bar{\mu }-K{\bar{\mu }}^{\left(\beta -1\right)},$$

which is the necessary condition for population persistence for population fluctuations between two fixed sizes [16]. Moreover, the inequality is valid for any distribution of population abundances following normal or log-normal distributions [16].

Rearranging the inequality we get:

(4) $$K^{\frac{1}{\left(2-\beta \right)}}<\bar{\mu }.$$

**Figure 1:**
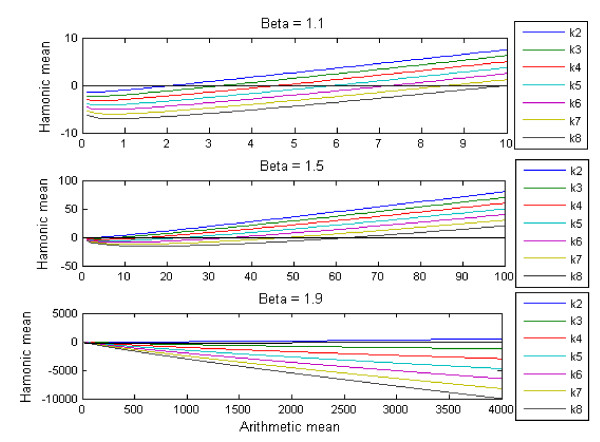
Graphical representation of the relationship between HM and $\bar{\mu }$ (see equation 2) for various values of *β *and *K*, with fixed *β *values of 1.1, 1.5 and 1.9 respectively, and with *K *varying from 2 to 8.

Thereby we obtained the lower boundary for the minimum population size necessary to avoid extinction through inequality (4). A graphical representation of the relationship between HM and $\bar{\mu }$ for various values of *β *and *K *is depicted in Fig. 1. For fixed *β *values of 1.1, 1.5, and 1.9 respectively, with *K *varying from 2 to 8 for each *β*, we see that for relatively small values of *β *= 1.1 the range of the lower boundary is relatively small, from $\bar{\mu }$ = 2 (*K *= 2) to $\bar{\mu }$ = 64 (*K *= 8). For intermediate values of *β *= 1.5 the range of the lower boundary increases from $\bar{\mu }$ = 1 (*K *= 2) to $\bar{\mu }$ = 64 (*K *= 8). For relatively high values of *β *= 1.9 the range of the lower boundary increases from $\bar{\mu }$ = 1 (K = 1) to $\bar{\mu }$ = 1024 (*K *= 2), $\bar{\mu }$ = 5.9 × 10^4 ^for (*K *= 3) and reaches extremely high values ($\bar{\mu }$ > 10^9^) for (*K *= 8) (see Fig. 1).

## Implications of the hypothesis

The methodology used will improve estimates of population viability and the fact that the parameters used in the model can be obtained from ordinary data sets gives this method a potentially wide applicability for comparing the chances of survival for fluctuating populations when facing environmental stochasticity. However, we note some of the potential difficulties associated with the estimation of the parameters of the model. The sampling variance or error in the measurement of population abundance, which generally scales directly with $\bar{\mu }$, would by itself produce a power-law slope of less than 2. This problem however is not important in data sets where the sample is large and thus sampling variance small.

The model is not taking into account the consequences of σ^2^_d_, which certainly affects the dynamics of populations. Such effects are, however, most prevalent for small populations. For larger population sizes, σ^2^_e _in *per capita *population growth rates leads to a variance that tends to scale with the square of the mean and hence overshadows the variance from σ^2^_d _which scales directly with $\bar{\mu }$. Hence, larger population size gives a better capacity of the model to predict the minimum viable population size. The model will therefore add to the ongoing debate about the relative importance of σ^2^_d _*versus *σ^2^_e _in determining the extinction risk. In fact we can deduce from the model that for certain values of *K *and *β *a population will become extinct even if its population size is sufficiently large to restrict the impact of σ^2^_d_. For the same reason we can see that a population can go extinct even if N_E _is high (and therefore harbour relatively high levels of genetic variability).

The fact that *β *is not only often different from 2 but also changes over time, has some implications for the association between population variability and extinction risk. Intuitively, for a given average abundance, one expects the risk of extinction to increase with temporal variability, however, many studies conducted on long-term data from natural populations have found the opposite result [2,17,18]. These studies use temporal variability as a direct proxy for population vulnerability, where population variability measures are calculated from time series data (std logN, CV). However, both the logarithmic transformation and the CV can be only properly applied if the variances scale proportionally to the square of the mean (*β *= 2), and given the fact that *β *is often different from 2 and is also changing with time it is quite evident that these correlation studies are potentially afflicted by this problem. The reasons for the discordant results obtained in these correlational studies have also been subject of a long debate and the relative importance of density dependent processes on population dynamics has been compared to the relative importance of environmental variability [19].

*β *is determined by the degree of correlation of the reproductive efforts among individuals within a population. In populations where the density is low we will expect a lack of correlation between reproductive efforts among individuals as the intraspecific interactions between individuals are very low, whereas if the population reaches higher density (for example when approaching the carrying capacity) we will expect a higher amount of interactions between individuals and an increased value of *β *which will increase the minimum population size necessary for avoiding extinction. Hence, a population near the carrying capacity with *β *near the value of 2 should be more prone to extinction, as when an environmental stochastic event is added *β *will become larger than 2, which means an increased risk of extinction. This finding could explain some of the metapopulation dynamics observed where a colonizing population initially increases in size only to suddenly go extinct. With environmental variability we do not only mean the amplitude of fluctuations of the environment but we also consider the spectrum of the fluctuations; one pole of this spectrum is 'white noise', where values of the variable are independent and variability entirely stationary, and at the other end of the spectrum there is the random walk or density-independent drift, for which the expected population variability grows at a rate proportional to the length of the series [20]. This effect has been linked to a reddened spectrum and there is increasing evidence that long-term ecological data sets showa reddened spectrum [21]. Inchausti & Halley (2002) concluded therefore that traditional measures of population variability need to be supplemented by a spectrum analysis.

The reddening of population dynamics has been suggested to be due to short-term fluctuations (of small amplitude) which are superimposed on ever larger long-period variations. However, the fact that *β *depends on the density of the population makes it quite evident that *β *and *K *(which are both influencing the variance of the population size) should be considered when interpreting the fluctuations of the population, and their changes in time should be taken into account when estimating the spectrum of the population dynamics. The need for diagnostic methods in population biology and conservation management undoubtedly becomes accentuated in the years to come [22]. In particular there will be a need for detecting regime shifts in the dynamic behaviour of populations as changes of the global environment begin to accelerate. This model allows an estimation of the importance of σ^2^_e _on the two parameters *β *and *K *and on how much alteration of the parameters will push the population towards the extinction threshold [23]. Environmental stochasticity will in fact increase or reduce the amplitude of the population fluctuations depending on the sign of the correlation between population size and environmental fluctuations as:

(5) σ^2^_tot _= σ^2 ^+ σ^2^_e _+ 2r(σσ_e_),

where σ^2^_tot _is the variance of the population size in presence of environmental noise, σ^2 ^is the variance of the population size in absence of environmental noise, σ^2^_e _is the environmental noise and r(σσ_e_) is the covariance between the environmental noise and the population fluctuation. The covariance is given by two times the product of r and the std of the population size and the environmental fluctuations (σ and σ_e _respectively). Hence, a negative correlation (r < 0) between environmental stochasticity and population fluctuations will decrease the fluctuations of the population size, with σ^2^_tot _< σ^2^, whereas in case of a positive correlation (r > 0) we will observe an increase in population fluctuations in the presence of environmental stochasticity (σ^2^_tot _> σ^2^).

## Abbreviations

$\bar{\mu }$: Arithmetic mean; CV: Coefficient of variation; r: Correlation coefficient; σ^2^_d_: Demographic stochasticity; N_E_,: Effective population size; σ^2^_e_: Environmental stochasticity; HM: Harmonic mean; log: Logarithm; *N*: Population size; std: Standard deviation; σ^2^: Variance.

## Competing interests

The authors declare that they have no competing interests.

## Authors' contributions

CP, LB and VL have conceived the study and drafted the manuscript. All authors have read and approved the final manuscript.

## Reviewers' comments

### Reviewer's report 1

Mark W. Schwartz, Department of Environmental Science & Policy, University of California, Davis Davis, CA 95616. Nominated by Peter Olofsson.

## Reviewer comments

This paper proposes using the harmonic mean of population to determine a minimum population size necessary to avoid extinction. This is equivalent to what conservation biologists have referred to as a 'minimum viable population'. In 1987 Michael Soule edited a book on Minimum Viable Populations. Subsequent to Soule's book there has been much literature on why the philosophical approach of estimating an MVP for conservation is inappropriate. Basically, MVP's encourage managers to manage to the lowest possible N. Mistakes in this realm being particularly costly. This, in fact, is exactly analogous to the problems with fisheries management. Promoting a measure that implies a bullet-proof estimate of a population size that is not threatened with extinction just begs for mistakes and failures if managers target/permit exploitation to reduce populations to those numbers. Thus, although Pertoldi and colleagues propose an advancement in terms of thinking parameters that may describe the minimum viable population, the very concept remains problematic with respect to management application.

### Author's response

*We agree with the Reviewer that the estimation of MVP for conservation purposes can be problematic and therefore we added some sentences to the Discussion section to point out these shortcomings. Furthermore, we explained how our model can make an important contribution to the ongoing debate about the changes of the extinction risk of populations in presence of different spectral noises*.

I am concerned that the HM can empirically incorrect owing to non-equilibrium dynamics in ecological systems. That harmonic mean estimates an extinction threshold assuming two things: that the dynamics driving the mean are at equilibrium, which they are often not, and that we actually know the variance, which we often do not. Both problems are particularly acute in species of conservation concern. To illustrate the latter, see Pimm (book, ~1995), who shows variance increasing through time as populations are further sampled. Sampling to estimate a mean routinely under-estimates the frequency of extreme events, and thus under-estimates extinction risk. Thus, our harmonic mean will increase through time and additional sampling. The fact that the drivers that affect the mean are assumed to be constant is a dangerous assumption illustrated by climate change. We simply can't assume, for management purposes, that we live in an equilibrium world. Things change, the mean changes with it.

### Author's response

*We agree that in natural populations equilibrium situations are rather the exception than the rule. However in populations with a long generation time it is possible to assume that the populations have a relatively stable dynamics, at least in the time frame that is interesting from a conservation perspective (which is typically not longer than a century). In the Discussion section we also mentioned how changes of β and K with increasing or decreasing population size could affect the spectrum of noise, suggesting new ideas for future studies. Lastly we also discussed how deviations of β from 2 can invalidate the comparisons of population variability where the coefficient of variation or the logarithm of the standard deviation have been used*.

Finally, this model appears to assume that populations are stable and near an extinction threshold. Imagine a population that is clearly on the brink of extinction. Surveys find, 4, 8, 6, 4, 12, 10 breeding females over a series of generations. The arithmetic mean of these numbers is 7.3, the harmonic mean is 6.15. I don't think that these authors, or any responsible conservation biologist, would want to be placed in a position of arguing that a harmonic mean of a population that was already in dire danger of extinction was secure with 7 breeding females. Rather, we would favor an interpretation that if inbreeding depression or demographic stochasticity doesn't doom this population, then catastrophic events (e.g., from hurricanes to habitat loss) surely will.

### Author's response

*We agree with the Reviewer that with small population size demographic stochasticity is playing a big role for the probability of extinction. Therefore both in the Introduction and the Discussion sections we discussed the concept of demographic stochasticity and we made clear that our model will only be applicable to populations which are large enough, i.e. populations where demographic stochasticity is not a major concern for their persistence. A reference dealing with the debate on the importance of density dependent processes on population dynamics compared to environmental variability has been added*.

### Reviewer's report 2

Josef Bryja, Department of Population Biology, Institute of Vertebrate Biology AS CR, 675 02 Studenec 122, Czech Republic. Nominated by Aniko Szabo

It seems to me that the paper may contain results that is worth publishing as it presents a novel approach which can be important for many scientific fields; Macroecology, Conservation Genetics and Population Ecology. The paper is overall well written and the maths are simply and clearly expressed. The authors do not explain all mechanisms that impact N_e_, but I guess that it is because of limited space. If some factors impact both β and N_e_, we expect things to be more complicated. It does not mean that the authors work is less relevant but, to the opposite, it open new perspectives that should be discussed. I think the introduction is sometimes difficult to follow due to the fact that the authors refer to their results in the introduction. Thank you for the opportunity to read about your inspiring work.

### Author's response

*We thank the Reviewer for comments and have modified the Introduction according to the suggestions given*.

### Reviewer's report 3

Wai-YuanTan, The University of Memphis Department of Mathematical Sciences. Memphis, TN 38152 United States

The authors assume that the mean and the variance will determine the pattern. This is equivalent to assume normal probability distributions for the random variables. This may be true in some cases but may not be so in other cases. The authors should provide strong evidence to just this. (Simply to quote one paper may not be enough). Aside from the above, I do not have any objection to the paper.

### Author's response

*We decided to add the following sentence and references to the MS following the Reviewer's comments: "Population size is expected to follow a log-normal distribution (which becomes normally distributed if log-transformed), given that it is the product of temporally multiplicative renewal processes *[13-15]. *The assumption of the model (a normal or log-normal distribution) is valid both for populations under exponential growth or decline and for populations at equilibrium *[13]. *It has been demonstrated in a survey of 544 long-term time series of terrestrial and aquatic organisms that about one-half of them are log-normally distributed, which implicates that our model can be applied also to long-term time series *[15].*"*
